# Theoretical Prediction of Divalent Actinide Borozene Complexes

**DOI:** 10.3390/molecules29235815

**Published:** 2024-12-09

**Authors:** Naixin Zhang, Qunyan Wu, Jianhui Lan, Weiqun Shi, Congzhi Wang

**Affiliations:** 1 College of Nuclear Science and Technology, Harbin Engineering University, Harbin 150001, China; zhangnx@ihep.ac.cn; 2 Laboratory of Nuclear Energy Chemistry, Institute of High Energy Physics, Chinese Academy of Sciences, Beijing 100049, China; wuqy@ihep.ac.cn (Q.W.); lanjh@ihep.ac.cn (J.L.); 3 School of Nuclear Science and Engineering, Shanghai Jiao Tong University, Shanghai 200240, China

**Keywords:** actinides, boron cluster, borozene complex, chemical bonding, density functional calculations

## Abstract

The aromatic boron cluster B_8_^2–^ (*D*_7*h*_) has similar π bonding to C_6_H_6_, which is named “borozene”. The B_8_^2–^ ligand has been observed to stabilize monovalent Ln(+I) in *C*_7*v*_-LnB_8_^−^ (Ln = La, Pr, Tb, Tm, and Yb) borozene complexes. Low-valency actinide complexes have been reported more rarely, and B_8_^2–^ may be one of the potential ligands. Here, we report a theoretical study on a series of actinide metal-doping octa-boron clusters AnB_8_ (An = Pa, U, Np, and Pu). It was found that each species has both half-sandwich and chair-like structures. Except for PaB_8_, the half-sandwich structures of UB_8_, NpB_8_, and PuB_8_ are more energetically stable than the chair-like structures, and the half-sandwich clusters of AnB_8_ are found to be actinide(II) borozene complexes with the M^II^[B_8_^2−^] type. For each of the half-sandwich clusters, the B_8_^2−^ ligand has σ and π double aromaticity. Various bonding analyses of AnB_8_ confirm the covalent interactions between the doped actinide metals and the octa-boron clusters, which further stabilize the complexes and determine the relative stability of AnB_8_. As expected, these complexes show high bond dissociation energies, especially PaB_8_ with stronger Pa-B covalent bonds. These results demonstrate that the B_8_^2−^ doubly aromatic ligand is able to stabilize divalent actinides.

## 1. Introduction

Boron exists in various forms in nature, among which boron clusters have unique structural and chemical properties [1,2,3,4,5,6,7]. Typical structures of boron clusters include tubular [8], bowl (B_36_) [3], cage (B_40_^−^/B_40_, borospherenes) [2], and bilayer boron clusters (B_48_) [9,10]. In general, small boron clusters usually have planar or quasi-planar structures [4,6,11,12]. Planar boron clusters are usually stabilized by delocalized σ and π bonds on the plane [4,6,7]. The boron clusters of B_7_^−^, B_8_^−^, and B_9_^−^ have been studied by combining photoelectron spectroscopy (PES) and theoretical calculations, and these clusters have in common the presence of a central boron atom and a monocyclic molecular wheel. It was found that B_7_^3−^ (*C*_6*v*_), B_8_^2−^ (*D*_7*h*_), and B_9_^−^ (*D*_8*h*_) are doubly aromatic with six delocalized σ and six delocalized π electrons, and they have similar π bonds to C_5_H_5_^−^, C_6_H_6_, and C_7_H_7_^+^, respectively [13], which are collectively referred to as “borozenes” [14].

In the past three decades, boron clusters doped with metal atoms have been studied experimentally and theoretically [15,16,17]. Metal-doped boron clusters can be broadly divided into two categories, one is monometallic-doped and the other is polymetallic-doped boron clusters. Monometallic-doped boron clusters are dominated by transition metals, for example Co©B_8_, Ru©B_9_ [12], Ta©B_10_, and Nb©B_10_ [18]. AuB_10_^−^ has also been analyzed experimentally and theoretically [19]. In addition to transition metals, alkali [20,21], alkaline earth [22,23], and lanthanide and actinide metals [13,24] can also be doped into boron clusters. At present, a series of metal-doped boron clusters with B_8_^2−^ are reported [25,26,27,28,29]. The first reported B_8_^2−^ complex is AlB_8_^−^ [27], which has *C*_7*v*_ symmetry, and the neutral cluster AlB_8_ is also a borozene complex with *C*_7*v*_ structure. Different metals doped with B_8_^2−^ display different structures, for example, when Au and Bi are doped with B_8_^2−^, the metal position is located near the edge of the boron ring [25,30]. The recently reported PbB_8_ cluster is found to be a closed-shell borozene complex with *C*_7*v*_ half-sandwich structure [31]. Another borozene complex, CuB_8_^−^, is found to be highly stable, also with a half-sandwich structure, by photoelectron spectroscopy (PES) and theoretical study [29]. The lanthanide-doped octa-boron cluster LnB_8_^−^ (Ln = La, Pr, Tb, Tm, Yb) [13] has been studied by PES and theoretical calculations. From early to late lanthanides, the global minimum of LnB_8_^−^ changes from *C_s_* to *C*_7*v*_ structure, and the *C*_7*v*_ structures contain monovalent Ln(I). Very recently, the corresponding actinide borozene complex AnB_8_^−^ (An = Ac, Pa, Bk, Md, and No) [24] was theoretically investigated, and complexes with half-sandwich structure adopt a +1 oxidation state.

In fact, the corresponding studies on borozene complexes doped with actinide metals are still very limited. We previously predicted a series of actinide(III) borozene AnB_7_ complexes (An = Pa, U, Np, and Pu) [32]. These clusters are M^III^[B_7_^3−^]-type complexes and have *C*_6*v*_ half-sandwich structures with double aromaticity B_7_^3−^ ligands. Apart from the above works, other actinide borozene complexes have not been reported so far. This raises the question of whether the neutral actinide borozene complexes with B_8_^2−^ are viable. In this work, we assess the feasibility of actinide-doped octa-boron AnB_8_ clusters (An = Pa, U, Np, and Pu) using first-principles calculations. These clusters are found to be stable divalent An(+II) complexes. Therefore, the B_8_^2−^ doubly aromatic ligand provides the possibility to design stable actinide(II) complexes.

## 2. Results and Discussion

### 2.1. Optimized Structures of AnB_8_

**Figure 1:**
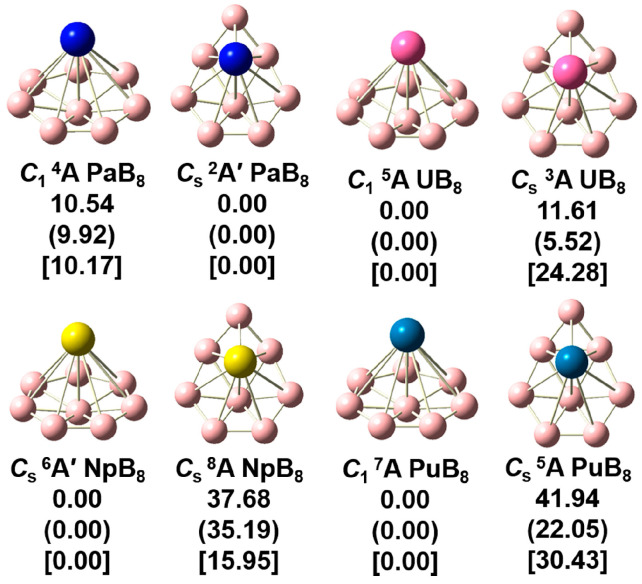
The half-sandwich and chair-like structures of AnB_8_ (An = Pa, U, Np, Pu) with the relative energies (in kcal/mol) at the PBE0/6-311+G*/RECP, TPSSh/6-311+G*/RECP (in parentheses), and CCSD(T)/cc-pVTZ (in medium parentheses) theoretical levels. Pink, indigo blue, magenta, yellow, and blue spheres represent B, Pa, U, Np, and Pu, respectively.

For UB_8_, the structures of the six low-lying isomers based on the global structure search were optimized at the PBE0/6-311+G* and TPSSH/6-311+G* theoretical levels. Figure S1 in the Supporting Information shows the optimized geometrical structures with the relative energies. The half-sandwich and chair-like configurations were found to be the two lowest isomers, and these two isomers were further evaluated at the CCSD(T)/cc-pVTZ theoretical level. Considering that the atomic radii of Pa, Np, and Pu are close to that of U, the structures of PaB_8_, NpB_8_, and PuB_8_ were individually optimized using the two low-lying isomers of UB_8_. For each isomer, different spin states were considered at the PBE0/6-311+G*/RECP and TPSSH/6-311+G*/RECP theoretical levels, for example, the double and quartet spin states of PaB_8_, the triplet and quintet spin states of UB_8_, the quartet and sextet spin states of NpB_8_, and the quintet and septet spin of PuB_8_. Figure 1 illustrates the optimized structures of half-sandwich and chair-like configurations of AnB_8_.

As shown in Figure 1, for PaB_8_, the half-sandwich structure is predicted to lie 10.54 kcal/mol (PBE0), 9.92 kcal/mol (TPSSh), or 10.17 kcal/mol (CCSD(T)) in energy above the chair-like structure. Except for PaB_8_, the half-sandwich structures of UB_8_, NpB_8_, and PuB_8_ are more energetically stable than the chair-like structures, and the energy differences are between 5.5 and 37.7 kcal/mol. As for the half-sandwich structures, the quartet, quintet, and septet structures are more stable for PaB_8_, UB_8_, and PuB_8_ with *C*_1_ symmetry, respectively, while the sextet structures with *C_s_* symmetry of NpB_8_ are more stable. Considering that the half-sandwich structures of the reported lanthanide complexes with B_8_^2−^ are low-valency complexes [13], in the subsequent discussions we only discuss the AnB_8_ clusters with the half-sandwich structures.

As shown in Table 1, the structures of AnB_8_ show very small spin contamination, i.e., the 〈S^2^〉 values are close to the corresponding ideal values of 3.75, 6.00, 8.75, 12.00. The atomic spin densities on Pa, U, Np, and Pu atoms are approximately 3, 4, 5, and 6, respectively, which are consistent with the spin states of AnB_8_. Thus, the unpaired electrons are mainly located on the actinide atoms, and these AnB_8_ clusters can be viewed as An^II^[B_8_^2−^]-type species. For the geometries of AnB_8_ at the PBE0/6-311+G*/RECP theoretical level, the calculated An-B_1_ (B_1_ is the central boron atom of the boron ring) bond distances range from 2.563 to 2.600 Å, which are slightly longer than the sum of the corresponding single-bond covalent radii of An and B [33]. In each AnB_8_ species, the average An-B_a_ bond distance (B_a_ refers to the peripheral boron atoms of the boron ring) is significantly longer than the An-B_1_ bond distance.

### 2.2. Bonding Nature of AnB_8_

To elucidate the charge transfer between actinide metals and the B_8_^2−^ ligands, VDD and Hirshfeld charges were calculated at the same level of theory. These charges are computed by performing spatial integration of the number of electron densities flowing into and out of atoms when forming chemical bonds. VDD charge analysis is not affected by the choice of functional and is primarily used to calculate charge flow between specific atoms or fragments. Hirshfeld charges are faster to calculate and are not based on the basis set. These charges are reliable for the analysis of charge transfer. Since the electronegativity of actinide elements is lower than that of boron atoms, the charge flow occurs from the actinides to the boron ligands. As shown in Table 2, the VDD charge values on the actinide metal atoms are all positive and range from 0.564 to 0.755, with the lowest value for NpB_8_. In the Hirshfeld charge analysis, the charge values of AnB_8_ are between 0.623 and 0.804, and NpB_8_ also has the lowest charge. These results indicate that the charge transfer in NpB_8_ is larger than those in other complexes. The charge rearrangement occurring in AnB_8_ may be influenced by the bonding interaction between the actinide metal cation and the B_8_^2−^ ligand. The WBIs of the An-B_1_ bonds range from 0.392 (PuB_8_) to 0.634 (PaB_8_). For each cluster, the WBI of An atoms and peripheral B_a_ atoms (An-B_a_ bond) is smaller than that of An-B_1_, in line with the shorter An-B_1_ bond distances, indicating that in the half-sandwich structures the bonding between the An atom and B_1_ atom is stronger than that of peripheral B_a_ atoms. From Pa to Pu, the WBIs of An-B_1_ and An-B_a_ bonds decrease gradually, indicating that the strength of the bonds is correspondingly weakened.

The singly occupied molecular orbital (SOMO) to lowest unoccupied molecular orbital (LUMO) gaps of the AnB_8_ clusters calculated at the PBE0/6-311+G*/RECP theoretical level are also listed in Table 2. As can be clearly seen from Table 2, the AnB_8_ clusters have the relatively large SOMO–LUMO gaps, which are 1.85–2.36 eV and 3.62–3.96 eV for the α-spin orbitals and β-spin orbitals, respectively. At the same theoretical level (PBE0/6-311+G*/RECP), the HOMO–LUMO gap of the optimized B_8_^2−^ ligand is 0.58 eV. The increased SOMO–LUMO gap of AnB_8_ indicates that actinide doping enhances the stability of the B_8_^2−^ ligand.

To further describe the specific components of bonding interactions, EDA was performed on the AnB_8_ complexes at the PBE0/TZ2P/ZORA theoretical level. The EDA analysis considered two types of fragments: the actinide cations (An^2+^) and the boron cluster (B_8_^2−^), as well as the neutral actinides (An) and boron cluster (B_8_). The interaction energy between the fragments consists of Δ*E*_elstat_, Δ*E*_Pauli_, and Δ*E*_orb_, as described by the equation of Δ*E*_int_ = Δ*E*_elstat_ + Δ*E*_Pauli_ +Δ*E*_orb_. Δ*E*_int_ represents the interaction energy between the two fragments. Δ*E*_elstat_, Δ*E*_Pauli_, and Δ*E*_orb_ denote the electrostatic interaction between the two fragments, the repulsive interaction due to Pauli repulsion effects, and the orbital interaction, respectively. Table 3 and Table S1 list the EDA results for AnB_8_. The percentages of Δ*E*_elstat_ and Δ*E*_orb_ represent Δ*E*_elstat_/(Δ*E*_orb_ + Δ*E*_elstat_) and Δ*E*_orb_/(Δ*E*_orb_ + Δ*E*_elstat_), which are used to express the electrostatic and covalent contributions of the two fragments, respectively.

As shown in Table 3, from PaB_8_ to PuB_8_, the absolute Δ*E*_int_ values show a decreasing trend with the fragments of An^2+^ actinide cations and B_8_^2−^ boron ligands. The absolute Δ*E*_elstat_ values gradually increase between −468.2 and −541.3 kcal/mol, which are more negative than the Δ*E*_orb_ values. This indicates the higher electrostatic interactions between the charged fragments of AnB_8_. PaB_8_ has higher Δ*E*_int_ and Δ*E*_orb_ energies than other complexes. Although PuB_8_ has the highest Δ*E*_elstat_ energies, it has the largest Δ*E*_Pauli_ energy, and PaB_8_ shows the lowest Δ*E*_Pauli_ energy. Therefore, the main contribution of Δ*E*_int_ for PaB_8_ mainly comes from the higher Δ*E*_orb_ interactions. Overall, for each cluster the contribution of electrostatic interaction Δ*E*_elstat_ (50.3–63.1%) is higher than that of orbital interaction Δ*E*_orb_ (36.9–49.7%). It should be noted that the trend of Δ*E*_elstat_ (%) is increased from to PaB_8_ to PuB_8_, which is different from the Δ*E*_int_ energies. This is mainly due to the Δ*E*_Pauli_ energies, which are increased from PaB_8_ to PuB_8_.

We also considered the interactions of PuB_8_ with the neutral An and B_8_ fragments (as shown in Table S1). The overall trends of the absolute Δ*E*_int_ values are same as those of the charged fragments (An^2+^ and B_8_^2−^), while the values are lower than those of the charged fragments, which may be due to the stronger electrostatic interactions of the charged fragments. The orbital contributions are in the range of 62.2–75.4%, which is higher than those with An^2+^ and B_8_^2−^ fragments (36.9–49.7%). Additionally, the Δ*E*_Pauli_ energies are higher than those with the charged fragments. The Δ*E*_orb_ energies are between −373.6 and −539.2 kcal/mol, which are significantly higher than the Δ*E*_elstat_ energies. These results suggest that for each cluster the Δ*E*_orb_ energy is the main contribution of the interaction energy.

**Figure 2:**
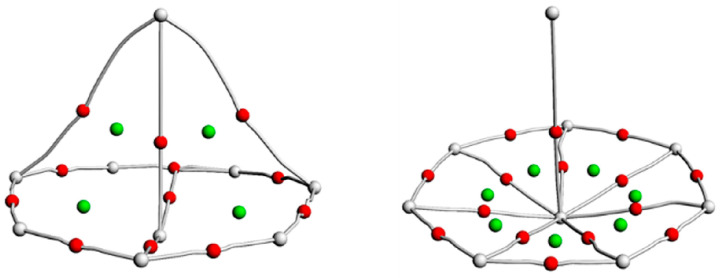
The QTAIM analysis of PaB_8_, UB_8_ (**left**) and NpB_8_, PuB_8_ (**right**) at the PBE0/6-311+G*/RECP level of theory. Red points represent bond critical points, grey lines represent bond paths, and green points represent ring critical points.

For further analysis of the bonding properties, the QTAIM topological analysis was performed at the PBE0/6-311+G*/RECP theoretical level. In the QTAIM analysis, the energy density (H), electron density (ρ), and Laplacian of the electron density (∇^2^ρ) are used to describe the bonding properties at the bond critical points (BCPs) between two atoms. As shown in Figure 2, PaB_8_ displays three Pa-boron BCPs including one Pa-B_1_ and two Pa-B_a_ bonds. Similarly, UB_8_ also shows three Pa-boron BCPs. In NpB_8_ and PuB_8_, only the An-B_1_ BCP is found. These An-B BCPs suggest the presence of metal–boron bonding interactions in AnB_8_. Table S2 lists the calculated ρ, ∇^2^ρ, and H values at the An-B BCPs. As shown in Table S2, the ρ values are lower than 0.1 a.u., which are between 0.04 and 0.05 a.u., and the ∇^2^ρ values range from 0.06 to 0.11 a.u., indicating the ionic metal–ligand bonding of the AnB_8_ clusters. The energy density (H) values are all negative and range from −0.008 to −0.012 a.u., suggesting partial covalency of these bonds.

To measure the degree of electron localization, the electron localization function (ELF) was also analyzed. Higher ELF values correspond to greater electron localization, indicating a stronger covalent character between the atoms involved. As shown in Table S2, the Pa-B bond exhibits a relatively higher ELF value, and the values decrease from Pa to Pu, suggesting that PaB_8_ has higher degree of electron localization and higher covalency. At the same theoretical level, delocalization index (DI) analysis was also performed for AnB_8_. The calculated total DI (DI_total_) values can be used to assess chemical bonding. As shown in Table S2, PaB_8_ has the largest DI_total_ value, indicating stronger covalent interactions in the AnB_8_ complexes. The DI_total_ values decrease sequentially from PaB_8_ to PuB_8_, which suggests the weakest covalent bonding of PuB_8_.

**Figure 3:**
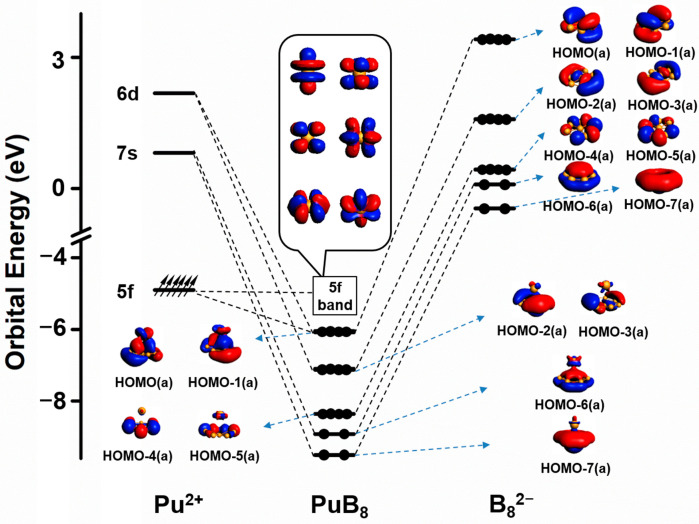
Orbital interactions of PuB_8_ at the PBE0/TZ2P/ZORA level of theory.

To gain deeper insight into the electronic structures of the AnB_8_ complexes, Kohn–Sham molecular orbitals (MOs) were plotted using the PBE0 method. The AnB_8_ clusters have open-shell electronic configurations, and their molecular orbitals (MOs) are shown in Figures S2–S5. PaB_8_ has three unpaired electrons, while UB_8_, NpB_8_, and PuB_8_ have four, five, and six unpaired electrons, respectively. These unpaired electrons correspond to the SOMO orbitals of AnB_8_, which are located on the actinide metal atoms. According to the MO analysis, the AnB_8_ clusters have relatively similar orbitals, and for each AnB_8_ cluster the HOMO and HOMO-1 orbitals are the An-B bonding orbitals, which mainly derive from the interactions between the 5f and 6d orbitals of the actinides and the 2p orbitals of boron atoms. Other MOs of AnB_8_ are skeletal bonding orbitals within the peripheral boron ring, primarily originated from B 2p atomic orbitals. With PuB_8_ as the representative, the orbital interaction diagram is shown in Figure 3. The HOMO(a) orbital of PuB_8_ is constituted by 13% Pu 6d and 6% Pu 5f orbitals, and HOMO-1(a) is also constituted mainly by Pu 6d and 5f orbitals. The low-lying orbitals from HOMO-2(a) to HOMO-7(a) originate mainly from MOs of B_8_^2−^, with small contributions from Pu 6d or 7s orbitals. The calculated NICS(0)_ZZ and NICS(1)_ZZ values of the B_8_^2−^ ligand in PuB_8_ are all negative (Table S3). In PuB_8_, HOMO(a), HOMO-1(a), and HOMO-6(a) orbitals are delocalized π bonds, while HOMO-2(a), HOMO-3(a) and HOMO-7(a) orbitals are assigned to delocalized σ bonds. Similarly, other clusters exhibit the same orbital characteristics (Figures S2–S5) and negative NICS(0)_ZZ and NICS(1)_ZZ values (Table S3). These results confirm that B_8_^2−^ in these clusters are doubly aromatic ligands. Therefore, similar to the half-sandwich lanthanide octa-boron LnB_8_^−^ clusters (Ln = La, Pr, Tb, Tm, Yb) [13], the studied half-sandwich An-doped clusters are borozene complexes with B_8_^2−^ doubly aromatic ligands.

**Figure 4:**
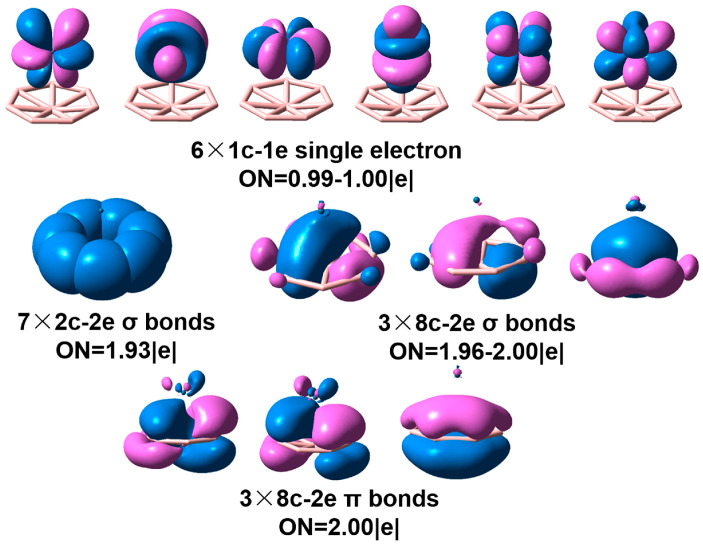
Bonding pattern of PuB_8_ from UAdNDP analysis with the occupation numbers (ONs) by the PBE0 method.

AdNDP analysis is a common method for studying the bonding characteristics in boron cluster species [34]. Since AnB_8_ are open-shell systems, unrestricted AdNDP (UAdNDP) analysis was conducted with PuB_8_ as the representative to further explore the chemical bonding properties of AnB_8_ (Figure 4). As illustrated in Figure 4, in line with Mulliken spin density analysis, PuB_8_ has six unpaired electrons (1c-1e) located on the 5f orbitals of the U atom, implying divalent Pu(II) in PuB_8_. In addition to the six unpaired electrons, the PuB_8_ complex possesses 13 pairs of valence electrons. According to UAdNDP analysis (Figure 4), 10 and 3 delocalized σ and π bonds are found in PuB_8_ with an occupation number (ONs) of about 2.0|e|, respectively. The 10 σ bonds consist of seven two-center two-electron (2c-2e) bonds located on the B_3_ triangle, and three eight-center two-electron (8c-2e) σ bonds on the quasi-planar B_8_^2−^ ring corresponding to the σ aromaticity of B_8_^2−^. PuB_8_ has three π bonds, which are eight-center two-electron (8c-2e) π bonds, and the first two π bonds confirm the Pu-B direct bonding interactions in PuB_8_.

### 2.3. Spectral Analysis

**Figure 5:**
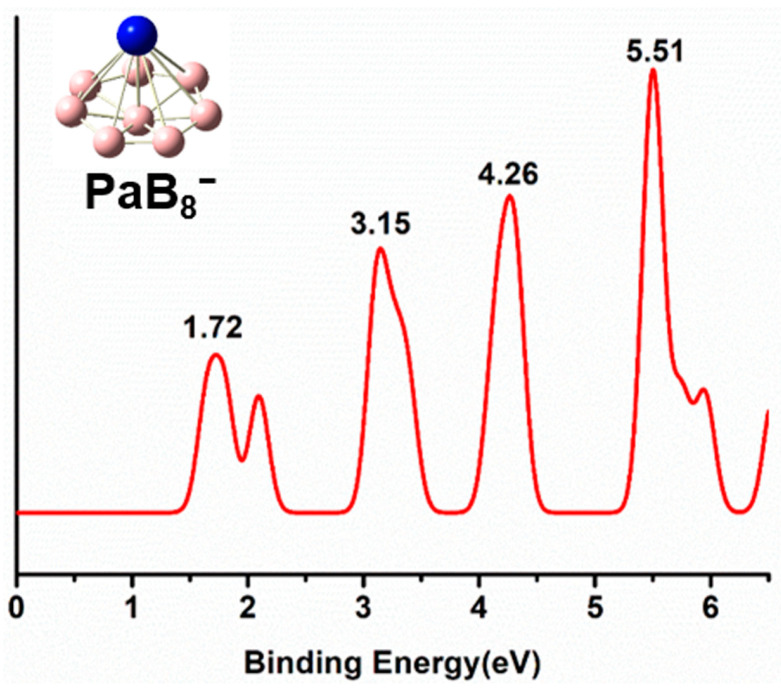
Simulated photoelectron spectrum of PaB_8_^−^ at the PBE0/6-311+G*/RECP level of theory.

Figure 5 and Figures S6–S8 shows the simulated PE spectra of the AnB_8_^−^ boron clusters at the theoretical level of PBE0/6-311+G*/RECP using the TD-DFT [35] method. The photoelectron spectra of AnB_8_^−^ clusters are similar with four characteristic peaks, indicating the similar structures and bonding of AnB_8_^−^ clusters. The first vertical detachment energy (VDE_1_) corresponds to ground state transition from AnB_8_^−^ to that of AnB_8_. The calculated VDE_1_ of PaB_8_^−^ is 1.72 eV, corresponding to removal of the 5f electron from the SOMO(a) orbital. As shown in Figure 5, PaB_8_^−^ exhibits a large energy gap between the first spectral band and the second band. After this large energy gap, there are three sharp and intense bands at 3.15, 4.26, and 5.51 eV. The simulated PES of UB_8_^−^ (Figure S6) is located at 2.19, 3.76, 4.92, and 6.20 eV. There are four main peaks of 2.89, 3.86, 4.37, and 5.70 eV in the PES of NpB_8_^−^ (Figure S7). For PaB_8_^−^, UB_8_^−^, and NpB_8_^−^, the relatively large energy gaps between the first band and the second band suggest the high electronic stability of PaB_8_, UB_8_, and NpB_8_. For the PES of PuB_8_^−^ (Figure S8), the energy gap is found to be smaller than other AnB_8_^−^ clusters, which implies the relatively lower stability of PuB_8_.

**Figure 6:**
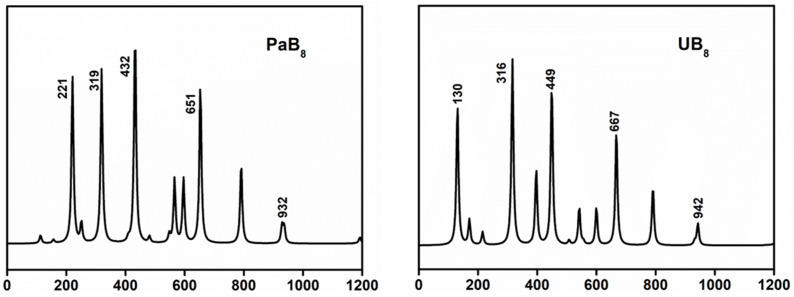
The infrared spectra of PaB_8_ and UB_8_ at the PBE0/6-311+G*/RECP level of theory.

The infrared (IR) spectra of the AnB_8_ boron clusters were calculated at the theoretical level of PBE0/6-311+G*/RECP. The IR spectrum of AnB_8_ shows the characteristics of the clusters, which is a fingerprint region used to identify the complex. The IR spectral peaks of AnB_8_ are mainly distributed from 100 to 1200 cm^−1^ and can be broadly divided into two regions: the low-frequency region (from 100 cm^−1^ to 800 cm^−1^) and the high-frequency region (from 800 cm^−1^ to 1200 cm^−1^), with the main characteristic peaks located in the low-frequency region. The vibrational modes of these main peaks include An-B bonds and B-B tensile vibrations. These vibrational modes are closely related to the molecular structures. This suggests that differences in actinide doping lead to differences in IR absorption in these regions. The calculated IR spectra of PaB_8_ and UB_8_ are shown in Figure 6. The IR peaks located at 319, 432, 651, and 932 cm^−1^ correspond to the vibrational peaks of the boron ring, and the characteristic peaks of B_8_^2−^ (360, 645, 938 cm^−1^) are maintained. The lower IR peaks at 221 cm^−1^ are attributed to the stretching vibration of the Pa atom and the B_8_^2−^ ring. For UB_8_, the vibrational peaks of the boron ring are located at 316, 449, 667, and 942 cm^−1^. The peak at 130 cm^−1^ is indicative of the interaction of U metal and B_8_^2−^. The NpB_8_ and PuB_8_ clusters show similar characteristic peaks (Figure S9). The calculated IR spectra demonstrate that the AnB_8_ clusters have distinct An-B interactions and B_8_^2−^ is maintained in each complex.

### 2.4. BDE Analysis of AnB_8_

The bond dissociation energies (BDEs) of the AnB_8_ clusters were assessed by considering the reactions of AnB_8_ → An + B_8_ and AnB_8_ → An^2+^ + B_8_^2−^. The structures of B_8_^2−^ and B_8_ were optimized at the PBE0/6-311+G*/RECP theoretical level, and B_8_^2−^ and B_8_ have planar structures. As shown in Table 4, these dissociation reactions are all endothermic with relatively larger positive BDE values, suggesting the high stability of these borozene complexes. For the BDEs with neutral charge products, the BDE value of PaB_8_ is 155.0 kcal/mol, which is the highest value in the species. This is consistent with the stronger covalent Pa-B bonding of AnB_8_. PuB_8_ shows the lowest BDE value in the species (97.6 kcal/mol). With respect to BDE trends, the stability of AnB_8_ gradually decreases from PaB_8_ to PuB_8_, which is in line with the trend of covalent interactions, indicating that it is mainly affected by the covalent interactions. As for the dissociation reactions of AnB_8_ → An^2+^ + B_8_^2−^, due to the stronger electrostatic interactions between the charged fragments of An^2+^ and B_8_^2−^, the overall BDE values are found to be larger (403.4–528.8 kcal/mol) compared to those with neutral fragments. These results indicate that dissociation reactions with neutral fragments are more likely to occur than those with charged fragments. Consistent with the covalent interaction of An-B bonds, the trend of BDE values also decreases from Pa to Pu, confirming that the covalent interactions determine the relative stability between these borozene complexes.

## 3. Methods

The global minimum structure search for UB_8_ was performed using the Crystal structure AnaLYsis by Particle Swarm Optimization (CALYPSO) program [36,37,38,39]. During the global structure search, 1000 sampled structures were assessed. Structural relaxation was performed in the Vienna ab initio simulation package (VASP) [40,41]. The structures of six low-lying isomers were optimized using the Gaussian 16 program package [42] with PBE0 and TPSSh methods, which are reliable methods for calculation of boron clusters [43,44,45]. For An, the quasi-relativistic effective core potentials ECP60MWB and the corresponding valence basis set ECP60MWB-SEG were used for An, and the 6-311+G* basis set was adopted for B. The optimized structures of the isomers and the relative energies are shown in Figure S1. In order to obtain more accurate relative energy, the two lowest isomers were further refined by the single-point coupled cluster CCSD(T) [46,47] calculation with the basis set of cc-pVTZ for B, and the relative energies are also shown in Figure S1. On the basis of the two low-lying isomers of UB_8_, the structures of PaB_8_, NpB_8_, and PuB_8_ were optimized at the theoretical levels of PBE0/6-311+G*/RECP and TPSSH/6-311+G*/RECP. All the structure calculations were carried out using Gaussian 16 program package.

At the PBE0/6-311+G*/RECP theoretical level, the photoelectron spectra (PES) of AnB_8_ were simulated using the Gaussian 16 program package with the time-dependent DFT (TDDFT) [35,48] method. The first vertical detachment energy (VDE) of AnB_8_ is calculated as the energy difference between the anionic ground state and the neutral ground state under the optimized anion geometry. The nucleus-independent chemical shift (NICS) [49] values were calculated at the same theoretical level by the gauge-independent atomic orbital (GIAO) method [50,51]. Hirshfeld charge [52], Voronoi deformation density (VDD) [53] charge, and quantum theory of atomic charges and atoms in molecules (QTAIM) [54] were obtained using Multiwfn 3.8 dev software [55,56]. Natural bond orbital (NBO) [57,58,59,60] analysis including Wiberg bond indices (WBIs) and unrestricted adaptive natural density distribution (UAdNDP) [34] analysis were calculated at the PBE0/6-311G*/RECP level of theory. Energy decomposition analysis (EDA) was performed using AMS2022 [61] at the PBE0/TZ2P theoretical level [62], taking into account scalar relativistic ZORA correction [63].

## 4. Conclusions

In this work, we have systematically analyzed a series of actinide metal-doped boron AnB_8_ clusters using first-principles calculations. Each AnB_8_ cluster has half-sandwich and chair-like structures, and the half-sandwich geometries are divalent actinide borozene complexes, which can be regarded as An^II^[B_8_^2−^]-type species. Since actinides are less electronegative than boron atoms, the charge flows from actinides to boron clusters. The PaB_8_ complex shows larger WBIs for Pa-B bonds and higher Δ*E*_int_, Δ*E*_orb_ energies, which is in line with the higher BDE. MO and AdNDP analyses confirm the An-B direct covalent bond interactions in AnB_8_ and the double aromatic ligand of B_8_^2−^. The calculated IR spectra of AnB_8_ show that these complexes have An-B interactions, and the B_8_^2−^ ligand remains in each cluster. According to the high BDEs, these complexes are all stable and the relative stability of AnB_8_ decreases from Pa to Pu, in line with the trend of the An-B covalent interactions. Therefore, the covalent interactions determine the relative stability between these borozene complexes. In addition, the dissociation reactions with the charged fragments are found to be more energetically favorable. This work is expected to improve our current understanding of the electronic structures of actinide borozene complexes and provide a theoretical basis for designing low-valency actinide complexes.

## Figures and Tables

**Table 1:** Spin states, spin contamination 〈S^2^〉, Mulliken atomic spin densities (ρ_An_), and average An-B_1_ and An-B_a_ bond distances (the B_1_ atom is the central boron atom of the boron ring, while the B_a_ atoms are the peripheral boron atoms of the boron ring) for AnB_8_ at the PBE0/6-311+G*/RECP level of theory.

Species	Spin States	〈S^2^〉	ρ_An_	Bond Distances* ^a^*	
				An-B_1_	An-B_a_
PaB_8_	Quartet	3.77	2.91	2.581	2.785
UB_8_	Quintet	6.02	3.93	2.563	2.790
NpB_8_	Sextet	8.78	5.15	2.600	2.854
PuB_8_	Septet	12.02	6.12	2.567	2.868

*^a^*: An-B_1_ is the bond of An to the boron atom at the center of the boron ring, An-B_a_ refers to the bond from An to the peripheral boron atom of the boron ring.

**Table 2:** The charge analysis of An, WBIs of the An-B bonds, and the SOMO–LUMO gap (eV) of AnB_8_ at the PBE0/6-311+G*/RECP level of theory.

Species	Atomic Charge		WBI		SOMO–LUMO Gap	
	VDD	Hirshfeld	An-B_1_	An-B_a_	α	β
PaB_8_	0.676	0.701	0.634	0.563	2.02	3.96
UB_8_	0.649	0.695	0.563	0.494	1.91	3.81
NpB_8_	0.564	0.623	0.456	0.446	1.85	3.86
PuB_8_	0.755	0.804	0.392	0.386	2.36	3.62

**Table 3:** The EDA results (kcal/mol) of AnB_8_ with An^2+^ and B_8_^2−^ fragments at the PBE0/TZ2P/ZORA level of theory.

Species	Δ*E*_int_	Δ*E*_Pauli_	Δ*E*_elstat_	Δ*E*_orb_	Δ*E*_elstat_ (%)	Δ*E*_orb_ (%)
PaB_8_	−777.8	152.4	−468.2	−462.0	50.30%	49.70%
UB_8_	−741.6	154.6	−475.7	−420.5	53.10%	46.90%
NpB_8_	−585.1	210.2	−502.1	−293.2	63.10%	36.90%
PuB_8_	−579.9	279.2	−541.3	−317.7	63.00%	37.00%

**Table 4:** Bond dissociation energy (kcal/mol) of AnB_8_ at the PBE0/6-311+G*/RECP level of theory.

Reactions	Pa	U	Np	Pu
AnB_8_ → An + B_8_	155.0	145.9	113.1	97.6
AnB_8_ → An^2+^ + B_8_^2−^	528.8	500.6	410.4	403.4

## Data Availability

Data are contained within the article or Supplementary Materials.

## Supplementary Materials

The following supporting information can be downloaded at: https://www.mdpi.com/article/10.3390/molecules29235815/s1, Figure S1: The low-lying isomers of UB_8_ along with the relative energies; Figures S2–S5: The MOs of PaB_8_, UB_8_, NpB_8_, and PuB_8_; Figures S6–S8: Simulated photoelectron spectrum of UB_8_^−^, NpB_8_^−^, and PuB_8_^−^; Figure S9: The infrared spectra of NpB_8_ and PuB_8_; Table S1: The EDA results (kcal/mol) of AnB_8_(An + B_8_); Table S2: QTAIM analysis of AnB_8_; Table S3: NICS values of B_8_^2−^ in AnB_8_; Tables S4–S11: Cartesian coordinates of AnB_8_.
