# Supplementary material for: Theoretical Prediction of Divalent Actinide Borozene Complexes
Source: Molecules. 2024 Dec 9;29(23):5815. doi: 10.3390/molecules29235815 (PMC11643500; doi:10.3390/molecules29235815)
Supplement: Supplementary file 1 [file molecules-29-05815-s001.zip › molecules-3314341-supplementary.pdf]

Theoretical Prediction of Divalent Actinide Borozene Complexes

Naixin Zhang [1,2], Qunyan Wu [2], Jianhui Lan [2], Weiqun Shi [1,2,3,*] and Congzhi Wang [2,*]

[1] College of Nuclear Science and Technology, Harbin Engineering University, Harbin 150001, China; zhangnx@ihep.ac.cn
[2] Laboratory of Nuclear Energy Chemistry, Institute of High Energy Physics, Chinese Academy of Sciences, Beijing 100049, China; wuqy@ihep.ac.cn (Q.W.); lanjh@ihep.ac.cn (J.L.)
[3] School of Nuclear Science and Engineering, Shanghai Jiao Tong University, Shanghai 200240, China
* Correspondence: shiwq@sjtu.edu.cn (W.S.); wangcongzhi@ihep.ac.cn (C.W.)

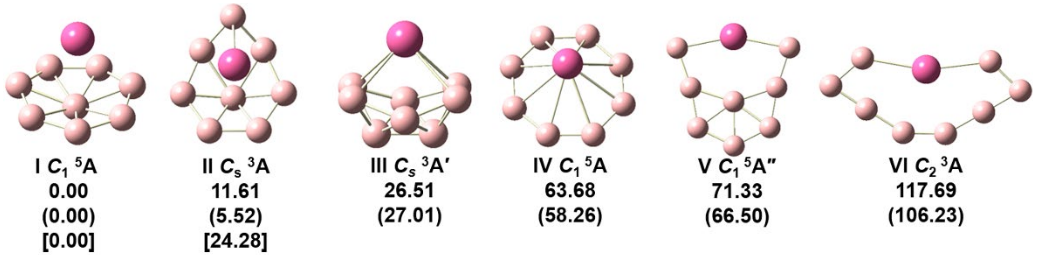

Figure S1. The low-lying isomers of UB_8 along with the relative energies (in kcal/mol) at the PBE0/6-311+G*/RECP, TPSSh/6-311+G*/RECP (in parentheses) and CCSD(T)/cc-pVTZ (in medium parentheses) theoretical levels. All the energies are corrected for zero-point energies. Pink, yellow, and magenta spheres represent B and U respectively.

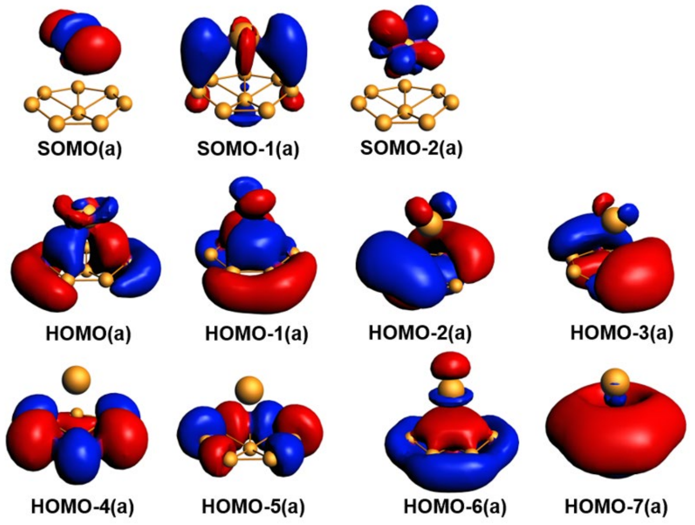

Figure S2. The MOs of PaB_8 at the theoretical level of PBE0/TZ2P/ZORA.

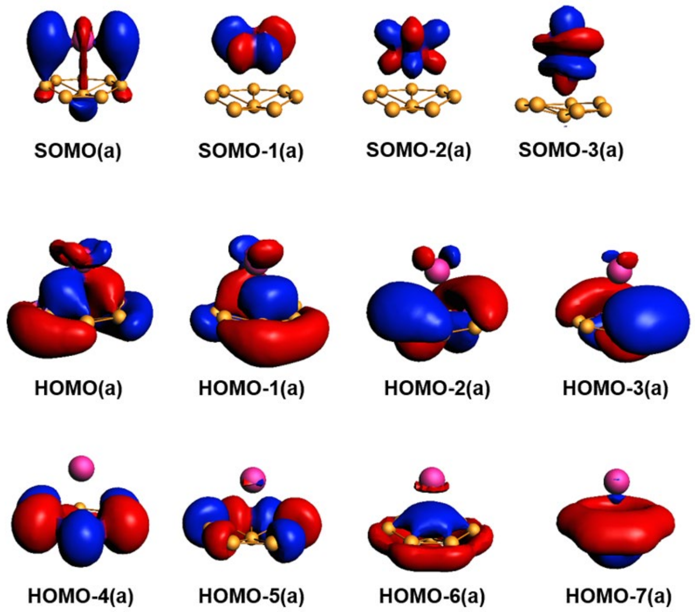

Figure S3. The MOs of UB_8 at the theoretical level of PBE0/TZ2P/ZORA.

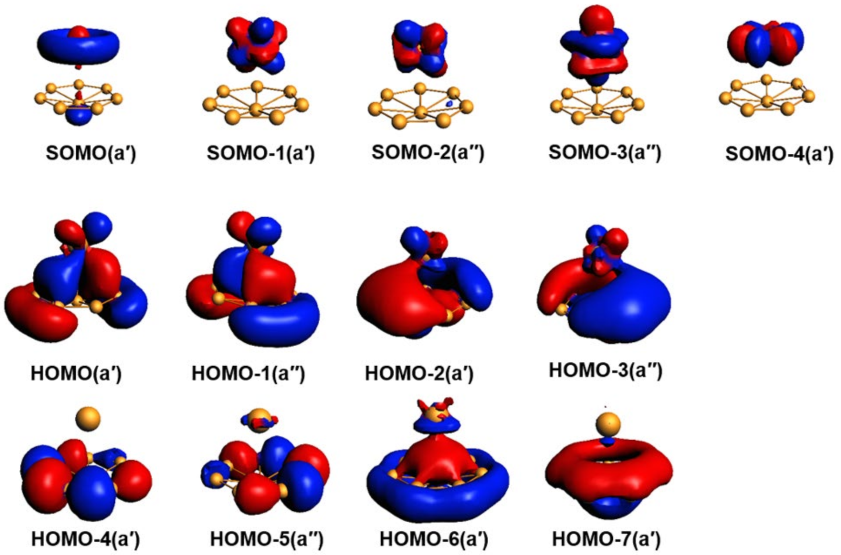

Figure S4. The MOs of NpB_8 at the theoretical level of PBE0/TZ2P/ZORA.

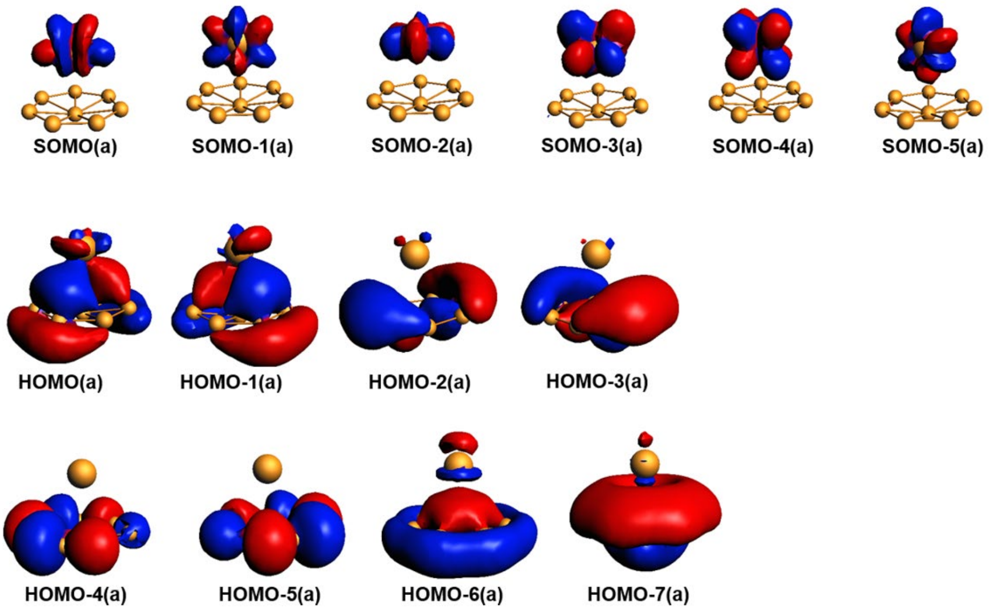

Figure S5. The MOs of PuB_8 at the theoretical level of PBE0/TZ2P/ZORA.

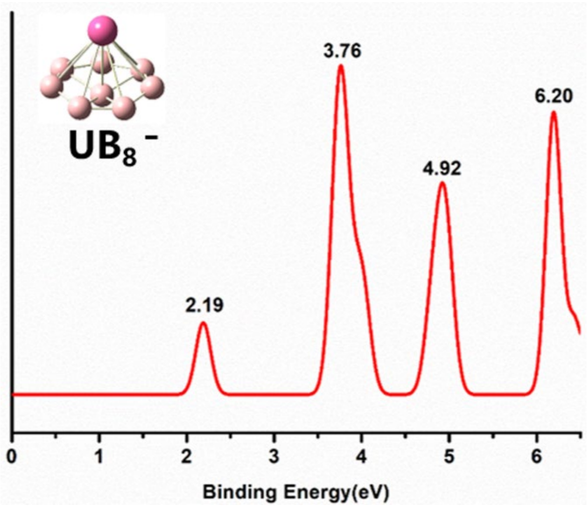

Figure S6. Simulated photoelectron spectrum of UB_8^- at the PBE0/6-311+G*/RECP level of theory.

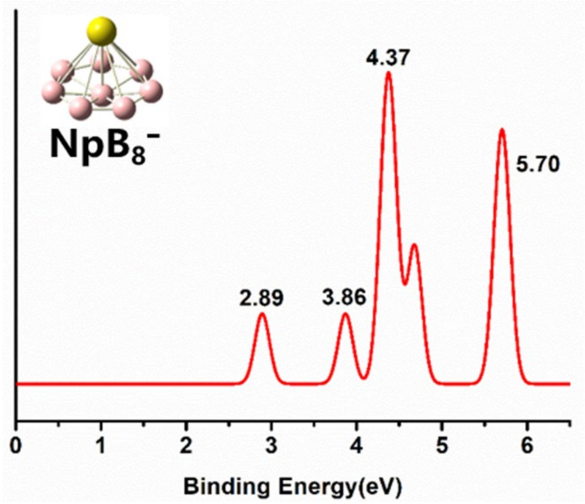

Figure S7. Simulated photoelectron spectrum of NpB_8^- at the PBE0/6-311+G*/RECP level of theory.

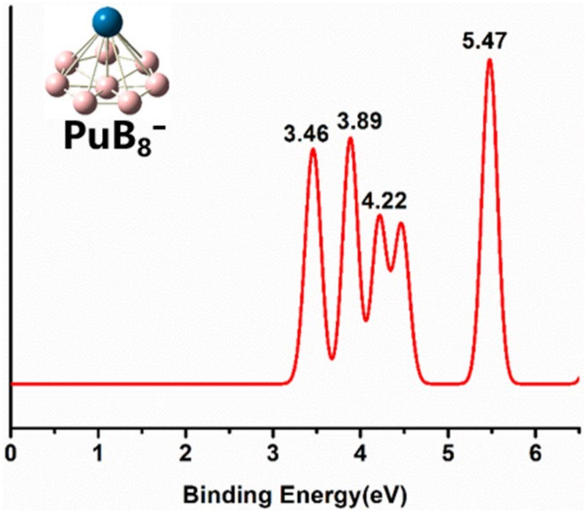

Figure S8. Simulated photoelectron spectrum of PuB_8^- at the PBE0/6-311+G*/RECP level of theory.

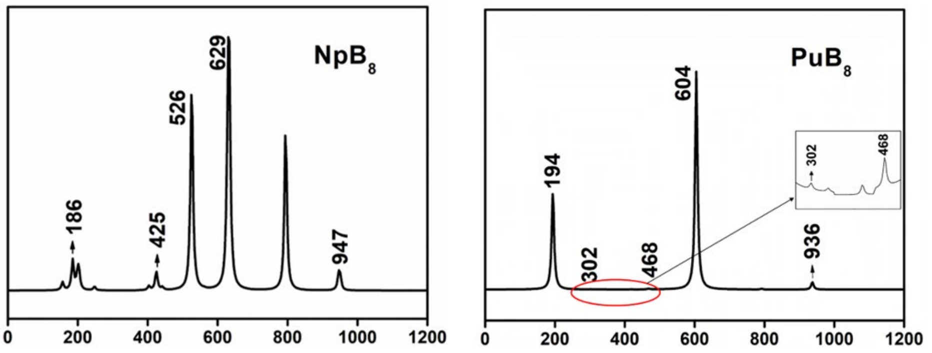

Figure S9. The infrared spectra of NpB_8 and PuB_8 at the PBE0/6-311+G*/RECP level of theory.

Table S1. The EDA results (kcal/mol) of AnB_8 (An + B_8) at the PBE0/TZ2P/ZORA theoretical level of theory. The values in parentheses indicate the percentage of each value relative to the sum of the values (ΔE_{elstat} +ΔE_{orb}).

Species	ΔE_{int}	ΔE_{Pauli}	ΔE_{elstat}	ΔE_{orb}	ΔE_{elstat}(%)	ΔE_{orb}(%)
PaB_8	−407.9	306.7	−175.5	−539.2	24.6%	75.4%
UB_8	−241.9	322.9	−191.1	−373.6	33.8%	66.2%
NpB_8	−−220.1	383.5	−222.3	−381.3	36.8%	63.2%
PuB_8	−210.8	427.9	−241.6	−397.0	37.8%	62.2%

Table S2. QTAIM analysis of AnB_8, density of electrons ρ, Laplacian of electron density $\nabla^2\rho$, energy density H, and the total delocalization index (DI_{total}) of the An-B bonds at the PBE0/6-311+G*/RECP theoretical level.

Species	ρ	H	$\nabla^2\rho$	ELF	DI_{total}
PaB_8	0.05148	−0.01177	0.06988	0.33499	2.848

UB_8	0.05016	−0.01123	0.08830	0.26826	2.741
NpB_8	0.04525	−0.00939	0.10922	0.17319	2.405
PuB_8	0.04410	−0.00894	0.10897	0.16524	1.970

Table S3. The calculated NICS(0)_ZZ and NICS(1)_ZZ values of the B_8^{2-} ligands in AnB_8 at the PBE0/6-311+G*/RECP theoretical level.

**Species**	**NICS(0)_ZZ**	**NICS(1)_ZZ**
PaB_8	−111.3	−46.6
UB_8	−203.8	−80.6
NpB_8	−126.6	−61.1
PuB_8	−28.8	−37.2

Table S4. The Cartesian coordinates of the optimized half-sandwich structure of PaB_8 at the PBE0/6-311+G*/RECP theoretical level.

**PaB_8**			
B	1.90635800	0.00127100	0.07585600
B	1.58783800	1.40086100	−1.05011100
B	1.43676300	0.76479900	1.61651300
B	1.58814200	1.39836600	−1.05215300
B	1.31327600	1.75171800	0.42197800
B	1.43896500	−0.76613100	1.61509700
B	1.65493700	0.00179600	−1.69150800

B	1.31766500	−1.75196300	0.41980900
Pa	−0.67274400	−0.00021900	−0.01953200

Table S5. The Cartesian coordinates of the optimized half-sandwich structure of UB_8 at the PBE0/6-311+G*/RECP theoretical level.

**UB_8**			
B	−1.89452900	0.00015800	−0.04081800
B	−1.53998700	1.39320400	1.07850600
B	−1.49318800	0.77119500	−1.59975200
B	−1.53999400	−1.39802500	1.07236700
B	−1.35420500	1.75176700	−0.40865300
B	−1.49373300	−0.76354300	−1.60294300
B	−1.62666000	−0.00379400	1.72010600
B	−1.35492900	−1.74973900	−0.41653600
U	0.66832700	−0.00006600	0.01074600

Table S6. The Cartesian coordinates of the optimized half-sandwich structure of NpB_8 optimized at the PBE0/6-311+G*/RECP theoretical level.

**NpB_8**			
B	1.91342200	0.01772100	0.00000000
B	1.55629800	−1.58056400	0.77013500
B	1.54969600	−0.37810200	−1.72785000

B	1.54969600	1.12220000	1.38508700
B	1.55629800	−1.58056400	−0.77013500
B	1.54969600	1.12220000	−1.38508700
B	1.54969600	−0.37810200	1.72785000
B	1.54209000	1.79066000	0.00000000
Np	−0.68639200	−0.00728200	0.00000000

Table S7. The Cartesian coordinates of the optimized half-sandwich structure of PuB_8 optimized at the PBE0/6-311+G*/RECP theoretical level.

**PuB_8**			
B	1.902298	−0.008431	0.077465
B	1.587218	1.345949	−1.113773
B	1.435777	0.836835	1.582693
B	1.564724	−1.459118	−0.990927
B	1.318433	1.774791	0.342035
B	1.426642	−0.697258	1.652159
B	1.647169	−0.086323	−1.692551
B	1.276407	−1.739138	0.500892
Pu	−0.668059	0.001796	−0.019670

Table S8. The Cartesian coordinates of the optimized half-sandwich structure of PaB_8 optimized at the TPSSH/6-311+G*/RECP theoretical level.

**PaB_8**			

B	1.90635800	0.00127100	0.07585600
B	1.58783800	1.40086100	−1.05011100
B	1.43676300	0.76479900	1.61651300
B	1.58814200	1.39836600	−1.05215300
B	1.31327600	1.75171800	0.42197800
B	1.43896500	−0.76613100	1.61509700
B	1.65493700	0.00179600	−1.69150800
B	1.31766500	−1.75196300	0.41980900
Pa	−0.67274400	−0.00021900	−0.01953200

Table S9. The Cartesian coordinates of the optimized half-sandwich structure of UB_8 at the TPSSH/6-311+G*/RECP theoretical level.

**UB_8**			
B	1.887135	−0.000066	−0.084878
B	1.542227	−1.404271	1.043847
B	1.412451	−0.768258	−1.615472
B	1.541994	1.403413	1.044790
B	1.215590	−1.764912	−0.426634
B	1.412714	0.769170	−1.615060
B	1.666594	−0.000641	1.678697
B	1.216391	1.765105	−0.425573
U	−0.646473	0.000025	0.021754

Table S10. The Cartesian coordinates of the optimized half-sandwich structure of NpB_8 optimized at the TPSSH/6-311+G*/RECP theoretical level.

**NpB_8**			
B	−1.892824	0.021270	0.000000
B	−1.411124	1.632569	0.770602
B	−1.529582	0.406583	−1.707720
B	−1.529582	−1.096998	1.373486
B	−1.411124	1.632569	−0.770602
B	−1.529582	−1.096998	−1.373486
B	−1.529582	0.406583	1.707720
B	−1.346669	−1.790553	0.000000
Np	0.654843	−0.006184	0.000000

Table S11. The Cartesian coordinates of the optimized half-sandwich structure of PuB_8 optimized at the TPSSH/6-311+G*/RECP theoretical level.

**PuB_8**			
B	1.865568	−0.006072	0.004718
B	1.533627	0.570052	1.686729
B	1.519847	−1.776049	0.187226
B	1.556239	1.513703	−0.931618
B	1.530072	−0.963371	1.502647
B	1.543594	−1.253479	−1.266740
B	1.544871	1.674170	0.603758

B	1.545306	0.209815	−1.762313
Pu	−0.672294	0.001661	−0.001298
